# Assessment of WHO 07/202 reference material and human serum pools for commutability and for the potential to reduce variability among soluble transferrin receptor assays

**DOI:** 10.1515/cclm-2022-1198

**Published:** 2023-04-18

**Authors:** Alicia N. Lyle, Jeffrey R. Budd, Victoria M. Kennerley, Bianca N. Smith, Uliana Danilenko, Christine M. Pfeiffer, Hubert W. Vesper

**Affiliations:** Division of Laboratory Sciences, National Center for Environmental Health, Centers for Disease Control and Prevention, 4770 Buford Hwy NE, MS S102-2, Atlanta, GA 30341, USA; Consulting Biostatistician, Shoreview, MN, USA.; Division of Laboratory Sciences, National Center for Environmental Health, Centers for Disease Control and Prevention, Atlanta, GA, USA.; Battelle, Columbus, OH, USA; Division of Laboratory Sciences, National Center for Environmental Health, Centers for Disease Control and Prevention, Atlanta, GA, USA.; Division of Laboratory Sciences, National Center for Environmental Health, Centers for Disease Control and Prevention, Atlanta, GA, USA.; Division of Laboratory Sciences, National Center for Environmental Health, Centers for Disease Control and Prevention, Atlanta, GA, USA.

**Keywords:** commutability, harmonization, reference material, soluble transferrin receptor, standardization, sTfR

## Abstract

**Objectives:**

The clinical use of soluble transferrin receptor (sTfR) as an iron status indicator is hindered by a lack of assay standardization and common reference ranges and decision thresholds. In 2009, the WHO and National Institute for Biological Standards and Controls (NIBSC) released a sTfR reference material (RM), 07/202, for assay standardization; however, a comprehensive, formal commutability study was not conducted.

**Methods:**

This study evaluated the commutability of WHO 07/202 sTfR RM and human serum pools and the impacts of their use as common calibrators. Commutability was assessed for six different measurement procedures (MPs). Serum pools were prepared according to updated CLSI C37-A procedures (C37) or non-C37 procedures. The study design and analyses were based on Parts 2 and 3 of the 2018 IFCC Commutability in Metrological Traceability Working Group’s Recommendations for Commutability Assessment. WHO 07/202 and serum pools were used for instrument/assay and mathematical recalibration, respectively, to determine if their use decreases inter-assay measurement variability for clinical samples.

**Results:**

The WHO 07/202 RM dilutions were commutable for all 6 MPs assessed and, when used for instrument calibration, decreased inter-assay variability from 208 to 55.7 %. Non-C37 and C37 serum pools were commutable for all 6 MPs assessed and decreased inter-assay variability from 208 to 13.8 % and 4.6 %, respectively, when used for mathematical recalibration.

**Conclusions:**

All materials evaluated, when used as common calibrators, substantially decreased inter-assay sTfR measurement variability. MP calibration to non-C37 and C37 serum pools may reduce the sTfR IMPBR to a greater extent than WHO 07/202 RM.

## Introduction

Iron deficiency is the leading cause of anemia and affects >2 billion people worldwide [1, 2]. Iron is crucial to physiologic functions including energy production and respiration; disruptions in iron homeostasis contribute to multiple disease pathologies [3]. Clinical causes and pathophysiological features of iron-deficiency anemia were reviewed previously [2, 4–6]. The measurement of a panel of serum-based iron status indicators is routinely used in clinical practice and in epidemiological studies, such as the National Health and Nutrition Examination Survey (NHANES) conducted by the Centers for Disease Control and Prevention (CDC) [7, 8].

Serum ferritin measurements are a clinically useful measure of iron storage, where low ferritin concentrations indicate iron deficiency [9]. However, ferritin can be affected by infection because inflammation increases ferritin concentrations, which is the subject of ongoing discussions [10]. Human transferrin receptor 1 (TfR1) is a homo-dimeric type II transmembrane receptor that, when cleaved at the extracellular domain, releases a soluble fragment known as sTfR [11–13]. TfR1 expression increases with iron deficiency to promote iron uptake [14]; thus, sTfR concentrations increase with iron deficiency and circulating sTfR concentrations parallel TfR1 numbers. sTfR levels are not influenced by acute-phase inflammation due to infection [7, 15]. In 2004, the joint WHO and CDC Technical Consultation on the Assessment of Iron Status at the Population Level (Geneva, April 6–8, 2004) concluded that the measurement of both ferritin and sTfR provides the best approach for estimating iron status in populations [9].

sTfR use in the clinical laboratory has been limited by analytical challenges attributed to a lack of assay standardization and common reference ranges and decision thresholds [7]. Furthermore, there is no reference measurement procedure for sTfR. In 2009, the WHO and National Institute for Biological Standards and Controls (NIBSC) developed a recombinant sTfR (rsTfR) reference material (RM), known as WHO/NIBSC 07/202, for the standardization of sTfR clinical measurement procedures (MPs) [16]. rsTfR was prepared by recombinantly expressing the human TfR gene (amino acids 121–760) in a lytic baculovirus/insect cell expression system [17]. The rsTfR generated was similar in structure to native, human sTfR, and was spiked into sTfR-depleted serum to generate the WHO 07/202 RM [18, 19].

Commutability is an essential RM property that indicates the material behaves like clinical samples (CS) when measured by two or more MPs [20]. RM commutability is important for assay calibration and ensures that clinical sample measurements are comparable across MPs irrespective of location and time of testing [21]. A common set of commutable serum samples or a commutable RM can be used to calibrate MPs to decrease inter-assay measurement variability [20]. In a 2010 study, dose-response plots demonstrated acceptable parallelism between the WHO 07/202 RM, manufacturers’ in-house standards, and three lyophilized serum samples [16]. Expressing serum sample sTfR content relative to WHO 07/202 improved agreement and reduced the geometric coefficient of variation for the three serum samples included in the study from 70–95 % to 13–19 % across 6 MPs [16]. However, a more robust study with a greater number of CS indicative of what would be encountered for patients, as well as a WHO dilution that falls within the CS concentration range, is needed to investigate the commutability of WHO 07/202 and to assess how the use of this material as a common calibrator impacts sTfR inter-assay measurement variability.

This study investigated the commutability of the WHO 07/202 sTfR RM across six commercial sTfR MPs, as well as the commutability of serum pools prepared according to updated CLSI C37-A procedures (C37) or using non-C37 procedures [22]. Commutability was assessed following the principles described in Parts 2 and 3 [23, 24] of the 2018 IFCC Commutability in Metrological Traceability Working Group’s Recommendations for Assessing Commutability [20, 23, 24]. WHO 07/202 was used for MP calibration and serum pools were used for mathematical recalibration to determine if the use of these materials as calibrators improves inter-assay clinical sample measurement agreement and decreases the inter-measurement procedure bias range (IMPBR).

## Materials and methods

### Serum pools

Solomon Park Research Laboratory (Kirkland, WA, USA) and BioIVT (Westbury, NY, USA) each prepared three serum pools with low, medium, and high sTfR concentrations. Serum pools from Solomon Park were prepared following the updated CLSI C37-A procedures [22] and serum pools from BioIVT used off-the-clot serum (see Online Supplement for details). Local IRBs approved sample collection protocols and documentation was reviewed and approved by the CDC Human Subjects Coordinator.

### Clinical samples

A panel of 20 commercially prepared, deidentified single donor sera were collected and processed by Solomon Park according to updated CLSI C37-A procedures and served as CS [22]. Samples were prescreened using the same MP used for NHANES measurements [25]. CS covered the “normal” sTfR concentration range of 1.65–5.40 mg/L, which corresponds to the central 95 % reference range in a healthy reference population of US non-pregnant women 15–49 years of age (NHANES 2003–2010) [25]. CS with high sTfR concentrations (>5.4 mg/L; 5/20 CS), low ferritin (<12 ng/mL; 6/20 CS), and/or borderline high c-reactive protein concentrations (>5 mg/L; 5/20 CS) were included to cover iron deficiency with and without the confounding effects of infection/inflammation.

### WHO 07/202 sTfR reference material

The WHO/NIBSC 07/202 sTfR RM is distributed by NIBSC (Hertfordshire, England) as a lyophilized preparation of rsTfR spiked into sTfR-depleted human serum [16, 17]. Each ampoule was reconstituted by the CDC Clinical Standardization Programs according to the manufacturer’s specifications. Briefly, ampoule contents were reconstituted in 0.50 mL of deionized water, yielding a WHO 07/202 stock with 21.7 mg/L (303 nmol/L) of rsTfR [16]. The stock was stored at −70 °C until shipment on dry ice to participants, who were instructed to store all materials at −70 °C until experimental use. Participants received one vial of WHO 07/202 stock per experimental run and were provided with instructions for how to dilute the stock on the day of the experiment to reach the MP calibration curve measurement range. Participants used specified volumes of WHO 07/202 and the diluent recommended by the assay manufacturer to generate six standards with the following concentrations: 2.7 mg/L (37.9 nmol/L), 2.17 mg/L (30.3 nmol/L), 1.8 mg/L (25.3 nmol/L), 1.35 mg/L (18.9 nmol/L), 1.1 mg/L (15.2 nmol/L), and 0.8 mg/L (11.7 nmol/L). All WHO dilutions were assessed for commutability. WHO 07/202 stability was tested internally for the 0.8 mg/dL (11.7 nmol/L) and 2.17 mg/dL (30.3 nmol/L) dilutions for two freeze-thaw cycles and confirmed as stable (see online Supplement for experimental details and Supplementary Table 1 for results).

### Experimental design

The CDC Clinical Standardization Programs provided all materials to study participants. Samples were shipped on dry ice and stored at −70 °C upon arrival. This study included eight MPs from five manufacturers (Table 1). Participants were asked to verify that the analytical system used was appropriately calibrated and performing according to manufacturer’s specifications by including internal quality controls during experimental runs. All materials were measured in triplicate in the same analytical run for two independent runs before and two independent runs after calibration using WHO 07/202. Instruments/assays were calibrated using WHO 07/202 according to internal, MP-specific protocols and procedures. Participants were instructed to perform all experiments using the same instrument, lots of reagents, calibrators, and controls.

### Data and statistical analyses

Data were submitted using an Excel template provided by the CDC Clinical Standardization Programs. Data were compiled and statistical analyses were performed using Microsoft Excel 2016 (Redmond, WA), the statistical add-in software Analyse-it version 4.97.4 (Leeds, UK), and R version 4.0.2. (Boston, MA). The mean, SD, and CV were calculated for each RM dilution, CS, and serum pool on each MP. MP-specific means were used for subsequent analyses.

### Commutability assessment

There is no sTfR reference measurement procedure. Commutability of WHO 07/202 and serum pools was assessed following the principles outlined in Parts 2 and 3 of the “IFCC Working Group Recommendations for Assessing Commutability” [23, 24]. Initial assessments were performed using the IFCC Part 2 difference in bias approach using a composite reference target, in line with an approach described previously [26]. The mean of S008–S010 was used as the composite reference target (‘mean target’) because measurements for the WHO 07/202 material from these MPs were within ±~20 % of the WHO 07/202 dilution values (Supplementary Table 2) as compared to ~75–230 % for the other MPs. The calibration effectiveness approach outlined in IFCC Part 3 was used to determine how the use of WHO 07/202 RM or serum pools as calibrators affects the IMPBR.

Bias plot assessments were performed (IFCC Part 2), where the y-axis was calculated as the difference between the ln-transformed MP-specific mean CS concentrations and ln-transformed mean target concentrations; the mean target values were plotted on the x-axis [24]. Bias limits (commutability criterion) were set based on sTfR biological variability, which was calculated according to Fraser [27]. The sTfR biological variability was calculating using a mean within-subject variability (CV_I_) of 6.9 % and a mean between-subject variability (CV_G_) of 19.1 %, as published in the European Federation of Clinical Chemistry and Laboratory Medicine’s (EFLM) database using previously published data [28–30]. The analytical performance specifications for desirable bias based on biological variability were calculated as 0.25 * √(CV_I_^2^ + CV_G_^2^) [27]. The acceptable difference in bias for RM vs. CS [bias limit] was set as the clinical sample mean bias ± biological variability. To assess commutability, the mean bias for each sample and MP were calculated and the bias range was derived by applying the related expanded measurement uncertainty. All calculations were done using ln-transformed data.

The impact of using common materials as calibrators on the inter-assay variability observed in CS, expressed as the IMPBR, was investigated using IFCC Part 3. For the WHO 07/202 RM, MPs were directly calibrated using the material, while mathematical recalibration was used for the serum pools [23]. Mathematical recalibration was conducted by performing regression analysis between the three C37 serum pool measurement results from each MP and the three C37 serum pool mean targets (mean of S008–S010). The intercept and slope of each MP-specific regression equation was then used with the original MP measurements to calculate the MP-specific measurements mathematically recalibrated to the C37 serum pools. This same procedure was applied using the three non-C37 serum pools to mathematically recalibrate to the non-C37 serum pools. For each CS, the mean concentrations were calculated on each MP before and after recalibration. For each MP, the percent difference between the mean for each CS and the mean target was calculated. The median percent difference across all CS was determined for each MP. The range between the highest and lowest MP CS median percent difference was calculated to find the IMPBR across all MPs.

## Results

### Initial data review

The assay characteristics for the eight sTfR MPs are listed in Table 1. MP sub-types included immunoturbidimetric, nephelometric, and enzyme-linked immunosorbent assays with unspecified traceability or traceability to WHO 07/202. For each MP, sTfR concentrations (mg/L) were measured in 20 CS, six WHO 07/202 dilutions, three C37 serum pools, and three non-C37 serum pools. S004 was excluded from further analyses because several CS, WHO 07/202 dilutions, and serum pool samples were below the measuring interval. S005 was excluded due to the low coefficient of determination (R^2^=0.424) from the regression fit between S005 measurements and the mean target values when compared to other methods (R^2^ range=0.9506–0.9978) and as reflected by high clinical sample scatter. Consequently, six MPs were included in the commutability assessments.

### Sample distributions across MPs

**Figure 1:**
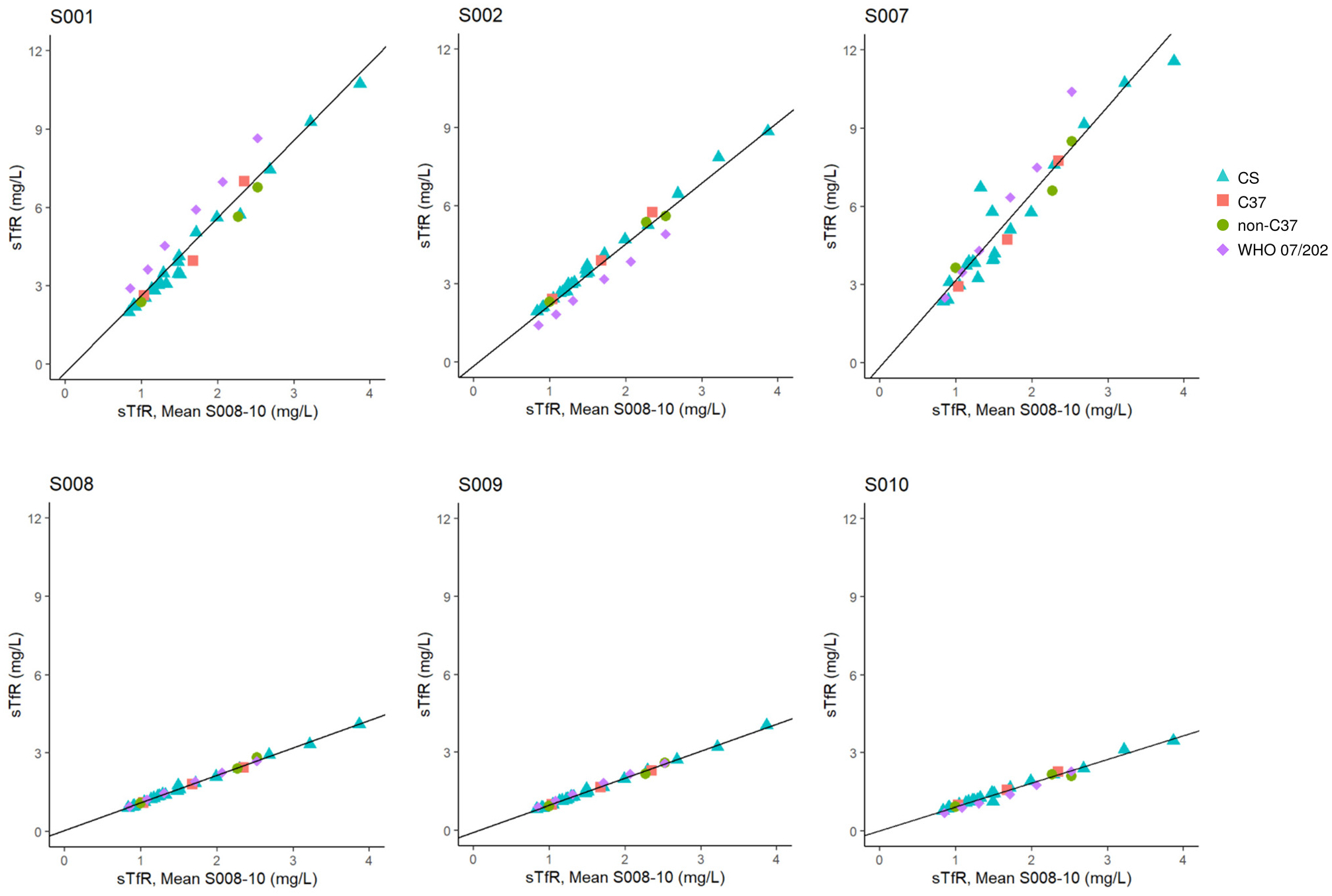
sTfR sample concentration distributions across measurement procedures. sTfR (mg/L) was measured for all individual clinical samples (CS; blue triangles), WHO 07/202 dilutions (purple diamonds), C37 serum pools (orange squares), and non-C37 serum pools (green circles) using the indicated measurement procedures (MPs). The mean of MPs S008–S010 was used as the target. MP-specific sample concentration distributions before standardization to a common material are plotted against the S008–S010 mean target.

MP-specific sTfR sample means before standardization were plotted against the S008–S010 mean target (Figure 1). IFCC Part 3 suggests using the trimmed mean as the target if CS results across MPs are normally distributed [23]. Because WHO material measurements from MPs S008–S010 agreed closely with WHO 07/202 RM dilution values (Supplementary Table 2), the mean of MPs S008–S010 was used as a mean composite reference target (‘mean target’) instead of the trimmed mean. The measurements for each MP for all CS included in the study showed good linear correlation with the S008–S010 mean target, with correlation coefficients from 0.9506 to 0.9978. Regression analysis slopes ranged from 0.911 to 2.969 and the intercepts ranged from −0.331 to 0.044 (Table 2).

### Assessment of bias patterns for materials and clinical samples

**Figure 2:**
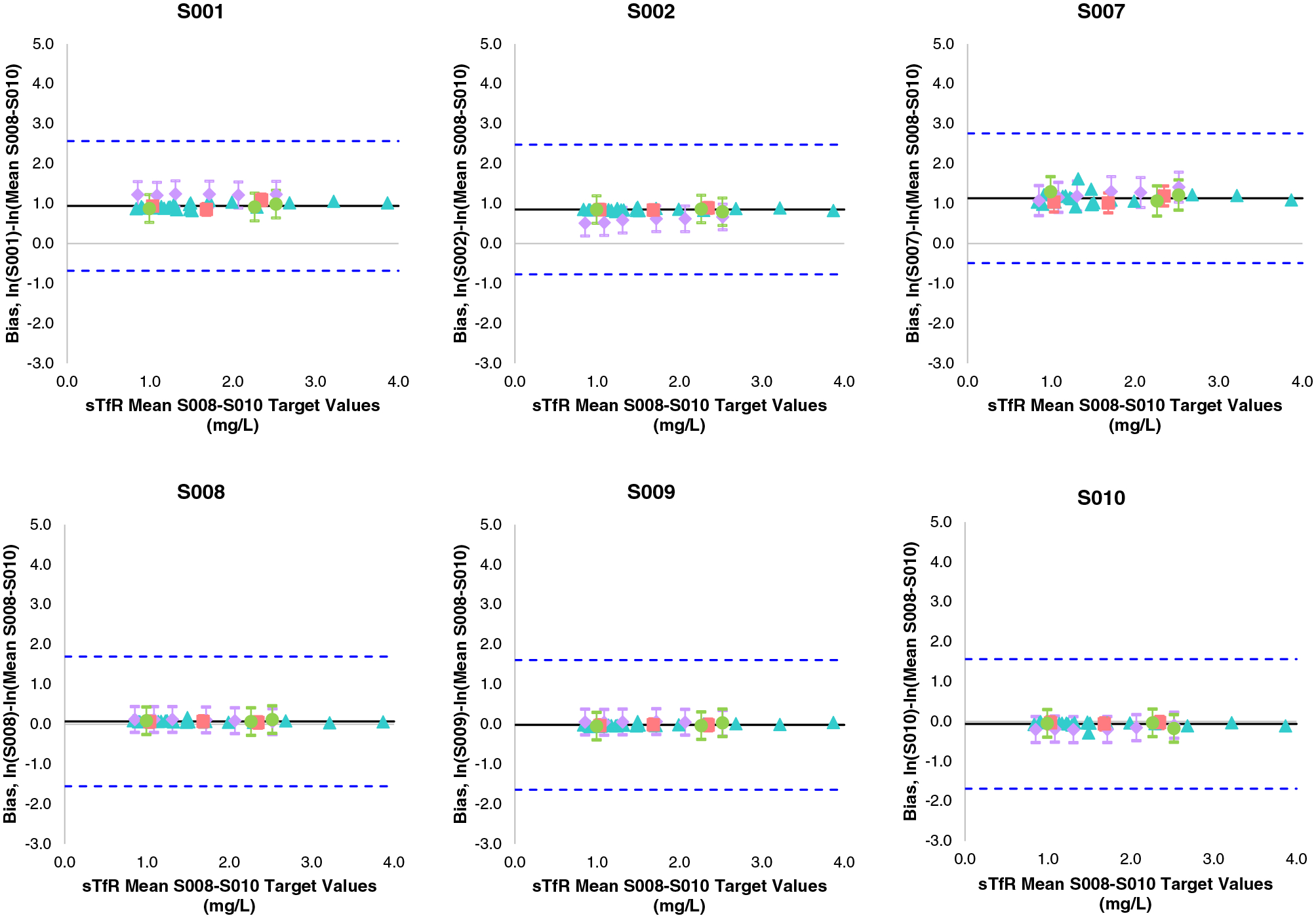
Bias patterns for the WHO 07/202, serum pools, and clinical samples for each measurement procedure. Bias plots were generated by plotting the calculated difference between the ln-transformed measurement procedure (MP)-specific mean sTfR concentrations (mg/L) and the ln-transformed S008–S010 mean target concentrations on the y-axis. The S008–S010 mean target concentrations were plotted on the x-axis. Clinical samples (CS; blue triangles), the WHO 07/202 dilutions (purple diamonds), C37 serum pools (orange squares), and non-C37 serum pools (green circles) are shown. Error bars show the expanded uncertainty of the difference in bias for each material assessed for commutability. Solid black line represents the mean CS bias between the MP measurements and the S008–S010 mean target; Blue dotted lines represent the bias limits (commutability criterion), as determined using the sTfR biological variability.

To obtain information about the commutability of WHO 07/202 and the C37 and non-C37 serum pools, bias plots were used and the material was deemed commutable if the mean bias and the bias ranges derived for each material from the expanded measurement uncertainty fell inside the bias limits (commutability criterion) (Figure 2; Supplementary Table 3). Bias limits were set as described in the Materials and Methods section. All WHO 07/202 RM dilutions assessed, as well as all C37 and non-C37 serum pools assessed, met the commutability criterion for all 6 MPs in the study (Supplementary Tables 3 and 4).

### Impact of MP calibration to the WHO 07/202 sTfR reference material

**Figure 3:**
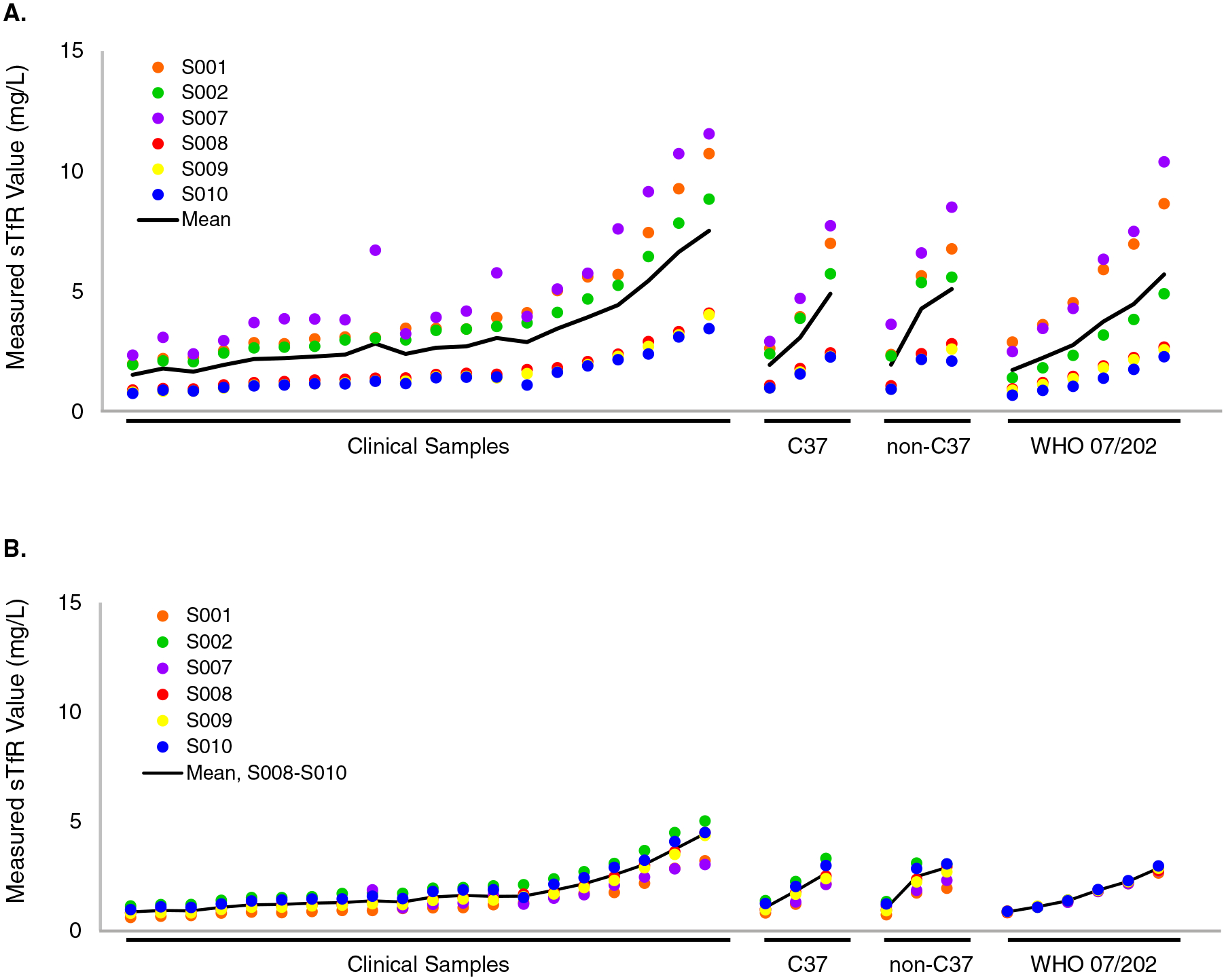
Sample concentration distributions across measurement procedures before and after calibration to the WHO 07/202 sTfR reference material. Samples are sub-grouped into clinical samples, C37 serum pools, non-C37 serum pools, and WHO 07/202 sTfR reference material dilutions and are plotted in order of increasing sTfR concentration. (A) Before standardization of the measurement procedures (MPs) to a common material, the sample distributions across MPs exhibited a bi-modal distribution. (B) After MP calibration using WHO 07/202 RM, the inter-assay sample distribution variability was decreased and exhibited a normal distribution pattern.

To determine the effectiveness of WHO 07/202 as a calibrator, manufacturers were asked to calibrate their MPs using WHO 07/202 according to internal protocols and procedures. The distributions of CS measurements across MPs before calibration using WHO 07/202 were highly variable and exhibited a bimodal distribution, as inferred from Figure 3A, with results from S008, S009, and S010 being clearly different from results obtained with the other MPs. Measurement results obtained on individual CS varied from 1.6 to 8.1 mg/L across MPs. CS variation improved to 0.5–2.0 mg/L after MP calibration using WHO 07/202 (Figure 3B).

**Figure 4:**
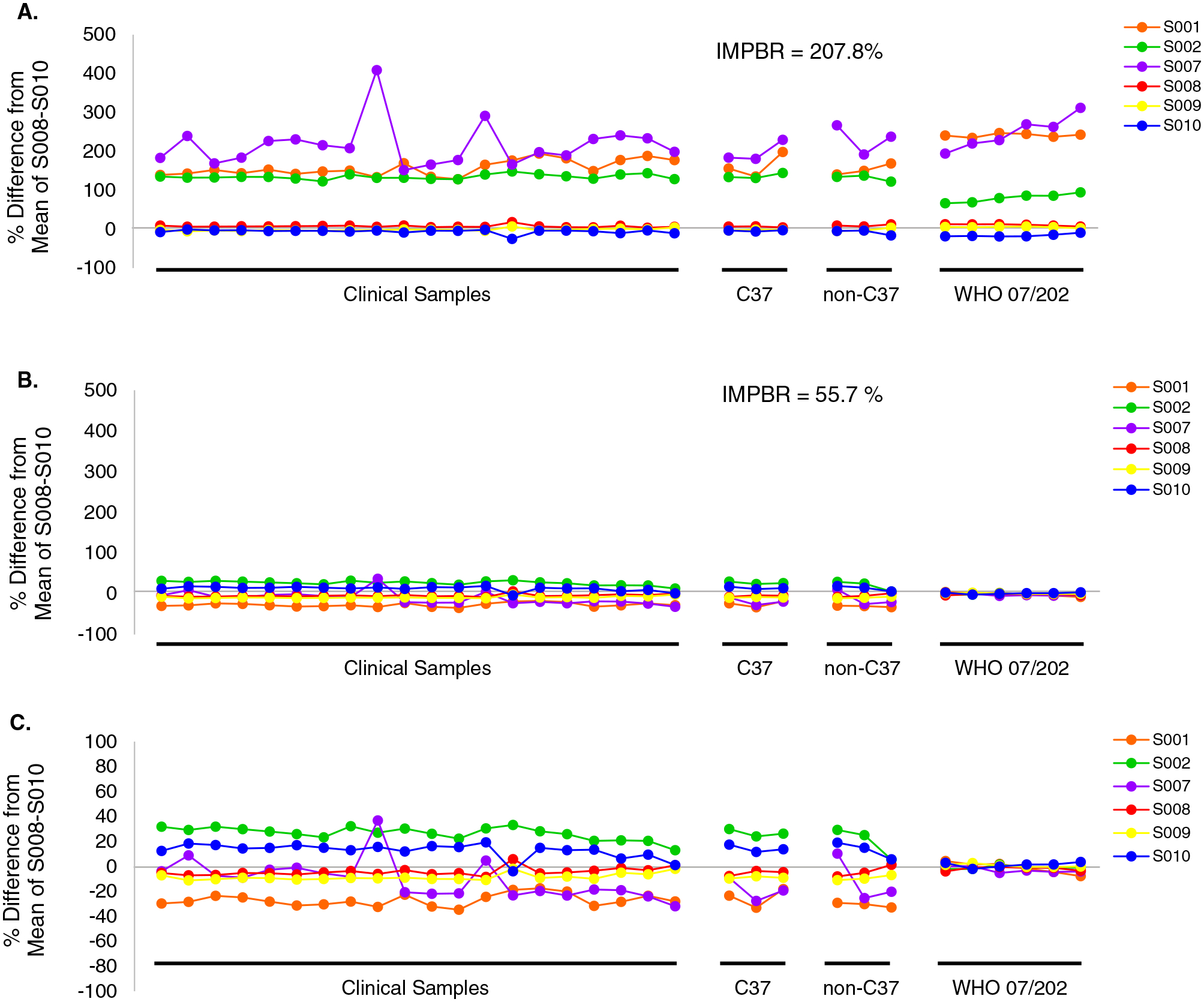
Percent differences from the S008–S010 mean target before and after calibration using WHO 07/202. Samples are sub-grouped into clinical samples, C37 serum pools, non-C37 serum pools, and WHO 07/202 RM dilutions and are plotted in order of increasing sTfR concentration. (A) The percent differences of the clinical samples, C37 and non-C37 pools, and the WHO 07/202 from the S008–S010 mean target were plotted and the inter-measurement procedure bias range (IMPBR) before standardization was 207.8 %. (B and C) After calibration using WHO 07/202, the IMPBR was reduced to 55.7 %. Panel B data are set to the same y-axis scale as panel A. Panel C data are the same as panel B with the y-axis scale changed for better data visualization.

To visualize MP-specific bias before and after calibration using the WHO 07/202 sTfR RM, the percent difference between the CS values and mean target were plotted before and after calibration. Before MP calibration, sTfR results exhibited an average CV of 47.9 % across CS and an IMPBR of 207.8 % (Figure 4A). S001 and S007 exhibited higher variability than other MPs, with standard deviations (SD) of 19.8 and 56.7 %, respectively, for the percent differences over all CS (Table 3). MP calibration using WHO 07/202 decreased the average CV across CS to 20.5 % and reduced the IMPBR from 207.8 to 55.7 % (Figure 4B and C), while also decreasing the S001 and S007 MP SDs to 4.8 and 15.6 %, respectively (Table 3).

### Impact of mathematical recalibration to C37 and non-C37 serum pools

**Figure 5:**
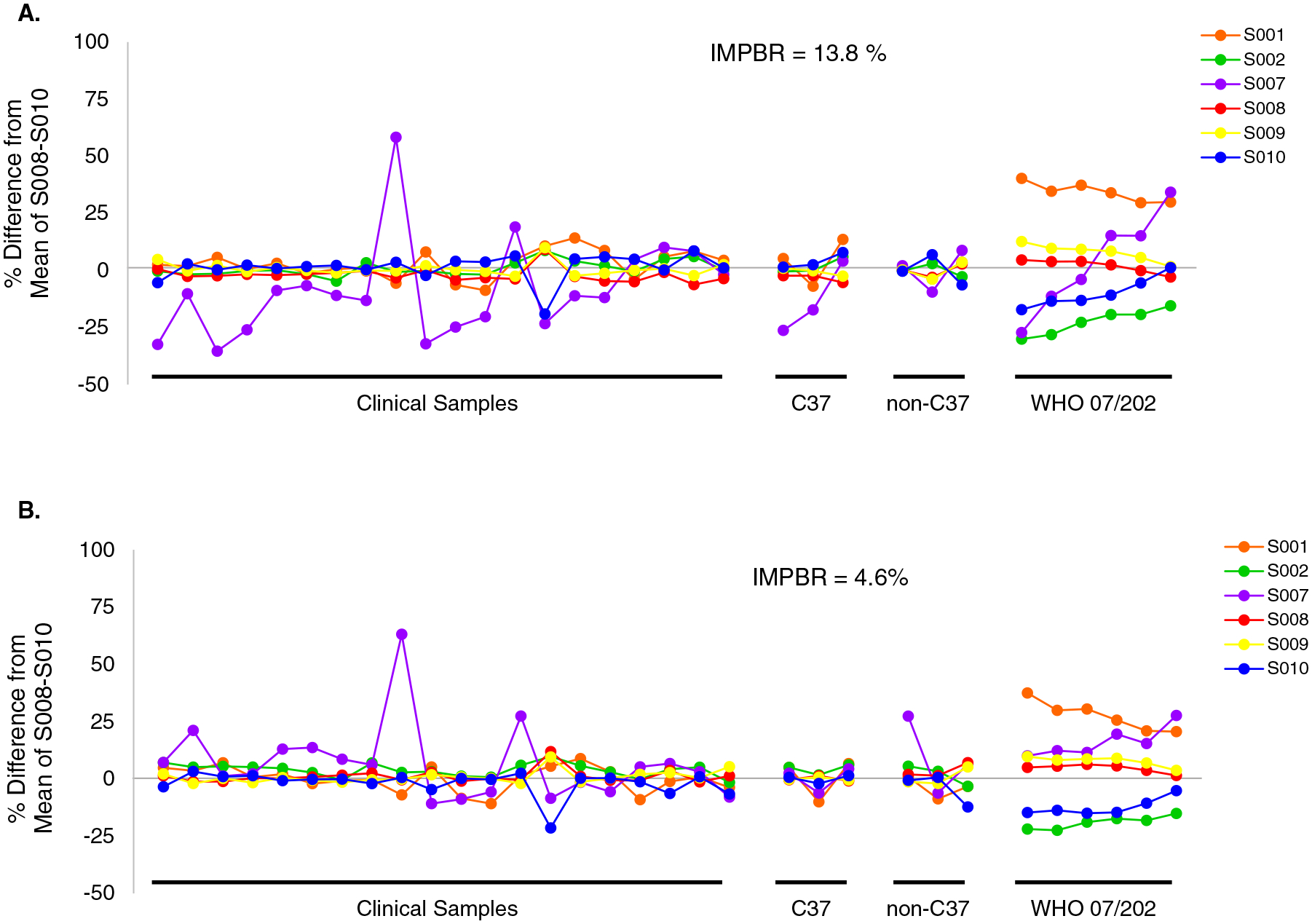
Sample percent differences from the S008–S010 mean target after mathematical recalibration of measurement procedures to C37 and non-C37 serum pools. Samples are sub-grouped into clinical samples, C37 serum pools, non-C37 serum pools, and WHO 07/202 RM dilutions and are plotted in order of increasing sTfR concentration. (A) The percent differences of the clinical samples, C37 and non-C37 pools, and WHO 07/202 from the S008–S010 mean target were plotted and the inter-measurement procedure bias range (IMPBR) was calculated after mathematical recalibration to non-C37 serum pools. The IMPBR was reduced from 207.8 to 13.8 %. (B) The percent differences of the samples from the S008–S010 mean target were plotted and the IMPBR was calculated after mathematical recalibration to C37 serum pools. The IMPBR was reduced from 207.8 to 4.6 %.

Mathematical recalibration to non-C37 serum pools reduced the average CV to 8.9 % and decreased the IMPBR to 13.8 % (Figure 5A), while also decreasing SDs for 4/6 MPs (Table 3). Mathematical recalibration to C37 serum pools reduced the average CV to 6.1 % and decreased the IMPBR to 4.6 % (Figure 5B), while also decreasing SDs for 5/6 MPs (Table 3). In contrast to the other MPs, S007 exhibited high sample-to-sample variability in bias initially and after all recalibration experiments. The IMPBR was reduced by mathematical recalibration of MPs to both the non-C37 (13.8 %) and the C37 (4.6 %) serum pools.

## Discussion

A clinician’s recommendations for patient care and disease management are based on established guidelines and laboratory test results. To use sTfR as a reliable iron status indicator, clinical laboratory measurements should be accurate over time and across locations and MPs. To achieve this, sTfR assays must be standardized to a commutable, higher order RM, such that inter-assay clinical sample measurements no longer vary by >200 %. This study evaluated the commutability of WHO 07/202 and C37 and non-C37 serum pools for potential use as common calibrators in the metrological traceability chain, which is an important aspect of standardization [20, 31]. In the absence of appropriate medical requirements, analytical performance specifications for desirable bias were derived from BV data. Study results demonstrated that WHO 07/202 dilutions are commutable for all 6 MPs assessed. Calibration of 6 MPs using WHO 07/202 profoundly improved inter-assay variability, reduced the IMPBR from 208 to 55.7 % (IMPBR decreased by 152.1 %), and established traceability of these MPs to the WHO RM. These substantial IMPBR improvements suggest that MP calibration using WHO 07/202 can considerably improve agreement among clinical measurement results. The use of non-C37 and C37 serum pools, all commutable for the 6 MPs in the study, for mathematical recalibration substantially improved inter-assay variability and reduced the IMPBRs to 13.8 % (non-C37) and 4.6 % (C37). The use of commutable reference materials as calibrators does not improve other aspects affecting analytical performance, such as MP selectivity, as indicated by the higher scatter observed with S007, which remains after recalibration, or lack of assay sensitivity, as observed with S005. Altogether, these data suggest that the use of non-C37 or C37-produced human serum pools for sTfR MP calibration, instead of the WHO 07/202 RM, would reduce the IMPBR to a greater extent. However, the ability of the non-C37 and C37 serum pools to harmonize MPs needs to be experimentally verified, for example, by using ISO 21151.

The commutability of the WHO 07/202 RM dilutions and the serum pools was initially assessed following the principles outlined in IFCC Part 2 and the impact of the use of these materials on IMPBR was assessed following the principles outlined in IFCC Part 3. In the absence of a reference measurement procedure, the use of the trimmed mean is recommended when the inter-assay data for CS are normally distributed [23]. In this study, the inter-assay data obtained for CS across all MPs were not normally distributed; therefore, the mean of MPs S008–S010 was set as a composite reference target and used in lieu of the trimmed mean, which is similar to an approach published recently [26]. The S008–S010 MPs were selected for this purpose because of the closeness of the WHO 07/202 measured values to the assigned WHO 07/202 values (Supplementary Table 2). An initial assessment of the impact of using the all-MP trimmed mean (IMPBR = 55.0 %; Supplementary Table 5) or the mean of MPs S008–S010 on the IMPBR (55.7 %; Table 3) suggest that very similar outcomes are achieved.

Similar to the study conducted by SJ Thorpe, et al. in 2010, our study found that sTfR commercial MPs showed poor agreement [16]. The 2010 study determined that WHO 07/202 reduced interlaboratory variation, when expressed as a percentage of the geometric coefficient of variation, to 12.8–18.8 % across three serum samples. In contrast, this study showed that calibration using WHO 07/202 only reduced the IMPBR to ~56 %. This study was more appropriately powered to fully assess WHO 07/202 RM commutability and the impact of the use of WHO 07/202 as a common calibrator and its ability to improve clinical sample measurement agreement across MPs.

We hypothesize that one potential reason the non-C37 and C37 pooled serum materials improved IMPBR over the WHO 07/202 RM is that serum pools contain native sTfR protein, while the WHO material contains recombinantly expressed sTfR generated in baculovirus/insect cells [16, 17]. While the rsTfR material is reported to form a stable dimer capable of binding two molecules of transferrin in a 2:2 stoichiometry, there are differences between the native sTfR protein in serum and the rsTfR protein in WHO 07/202 [13, 17]. The rsTfR in WHO 07/202 lacks 2 inter-chain disulfide residues (Cys-89 and Cys-98) and is N-glycosylated, features consistent with native sTfR [18, 19]. However, the rsTfR in WHO 07/202, when compared to native sTfR, is 20 amino acids shorter at the N-terminus and differs with respect to post-translational modifications, where rsTfR lacks 1 O-linked glycan at Thr-104 and may differ in N-glycosylation oligosaccharide content from the use of a baculovirus-insect cell expression system [19, 32]. It is possible that the lack of these features in the rsTfR in WHO 07/202 causes the material to be recognized differently by MPs when compared to native sTfR. Differences could also result from what epitope sequences are recognized by the polyclonal antibodies used by the MPs in this study. Without knowledge of the specific epitope sequences targeted, due to the proprietary nature of this information, it is difficult to know if antibody epitope and/or structural protein differences are causal factors for why WHO 07/202 and serum pools differ in their effectiveness to reduce inter-assay variability when used as common calibrators.

Strengths of this study, compared to previous assessments, include the use of CS that cover the sTfR concentration range and CS that would normally be encountered in the clinical setting, including samples indicative of iron deficiency with and without the confounding effects of infection/inflammation. These samples did not exhibit significant differences across sTfR MPs. Additional strengths include the use of diluted WHO 07/202 material. The WHO 07/202 material, when reconstituted according to NIBSC instructions, contains 21.7 mg/L (303 nmol/L) of sTfR. To be within the measuring intervals used for CS, the WHO 07/202 stock was diluted from 1:8 to 1:26 and all dilutions were evaluated for commutability.

Limitations of the study include that not all commonly used MPs on the market were included and that sTfR measurements were performed using a single lot of reagents and calibrators with the assumption that each clinical analyzer was performing and being operated according to manufacturers’ specifications. The conclusions about commutability are based on the following assumptions: 1) similar results would be obtained with different reagent and calibrator lots, 2) similar results would be obtained with different instrument operators and/or different instruments of the same type, and 3) similar results would be obtained in the future using the same clinical analyzers or clinical analyzers with equivalent performance.

## Supplementary Material

Supplement

**Supplementary Material:** This article contains supplementary material (https://doi.org/10.1515/cclm-2022-1198).

## Figures and Tables

**Table 1:** Description and selected characteristics of measurement procedures included in the study.

Assay manufacturer (Lab ID)	Analyzer	Assay name	Assay type^a^	Measuring interval	LOQ^b^	Stated traceability
Roche (S001)	Cobas^®^ c501	Tina-quant sTfR I	IA	0.5–40.0 mg/L	0.5 mg/L	In-house material
Roche (S002)	Cobas^®^ c501	Tina-quant sTfR II	IA	0.5–20.0 mg/L	0.4 mg/L	In-house material
Siemens (S004)^c^	Atellica CH930^®^	Latex-enhanced sTfR	IA	0.5–11.77 mg/L	0.5 mg/L	WHO RM 07/202
Quansys (S005)^c^	Q-View LS Imager^®^	Q-Plex Human Micronutrient (7-Plex)	ELISA	0.15–123 mg/L	0.15 mg/L	WHO RM 07/202
Ramco (S007)	N/A	Human transferrin receptor	ELISA	0.1–40 μg/mL	0.1 μg/mL	In-house material
Beckman (S008)	Access 2^®^ Enhanced	sTfR	IA	0.004–11.07 mg/L	0.004 mg/L	In-house material
Beckman (S009)	DxI 800^®^	sTfR	IA	0.004–11.07 mg/L	0.004 mg/L	In-house material
Siemens (S010)	BN Pro Spec^®^	N Latex sTfR	N	0.14–4.4 mg/L	0.144 mg/L	In-house material

a IA, immunoassay; N, nephelometry; ELISA, enzyme-linked immunosorbent assay;

b LOQ, limit of quantification;

c MPs were excluded from commutability analyses.

**Table 2:** Weighted Deming regression analysis comparing measurement procedure results against the mean target.

Assay	Lab ID	Intercept	95 % CI, intercept	Slope	95 % CI, slope	Correlation coefficient
Roche, Cobas c501 Tina-quant sTfR I	S001	−0.331	−0.869 to 0.208	2.969	2.671–3.266	0.9658
Roche, Cobas c501 Tina-quant sTfR II	S002	−0.139	−0.461 to 0.183	2.340	2.162–2.518	0.9799
Ramco, ELISA, human transferrin receptor	S007	−0.160	−0.898 to 0.578	2.837	2.934–3.748	0.9506
Beckman, Access 2	S008	0.044	−0.003 to 0.091	1 .052	1 .026–1 .078	0.9978
Beckman, DxI 800	S009	−0.060	−0.112 to −0.007	1 .038	1.010–1.067	0.9973
Siemens, BN ProSpec	S010	0.016	−0.076 to 0.108	0.911	0.860–0.961	0.9890

**Table 3:** Median percent biases and SDs across all clinical samples for each MP and inter-measurement procedure bias range across MPs – before and after standardization using the calibration effectiveness approach following the principles described in IFCC Part 3.

	S001	S002	S007	S008	S009	S010
**Before standardization**						
Median bias before standardization, %	151.4	133.5	203.5	6.8	−2.1	−4.3
SD before standardization, %	19.8	6.2	56.7	3.0	3.0	5.2
IMPBR before, %	207.8					
**After calibration to WHO 07/202**						
Median bias after standardization, %	−28.2	27.5	−13.8	−5.3	−9.3	14.6
SD after standardization, %	4.8	5.2	15.60	3.2	2.7	5.7
IMPBR after, %	55.7					
**After mathematical recalibration to non-C37 serum pools**						
Median bias after standardization, %	2.8	−0.2	−11.0	−2.5	0.0	2.2
SD after standardization, %	6.0	3.3	21.7	3.2	2.8	5.7
IMPBR after, %	13.8					
**After mathematical recalibration to C37 serum pools**						
Median bias after standardization, %	0.8	4.4	4.2	0.3	0.2	−0.2
SD after standardization, %	5.5	2.9	16.8	2.9	2.7	5.3
IMPBR after, %	4.6					

IMPBR, inter-measurement procedure bias range; MP, measurement procedure; SD, standard deviation.

## References

[1] Kassebaum NJ, Jasrasaria R, Naghavi M, Wulf SK, Johns N, Lozano R, A systematic analysis of global anemia burden from 1990 to 2010. Blood 2014;123:615–24. 24297872 10.1182/blood-2013-06-508325 PMC3907750

[2] McLean E, Cogswell M, Egli I, Wojdyla D, de Benoist B. Worldwide prevalence of anaemia, WHO vitamin and mineral nutrition information system, 1993–2005. Publ Health Nutr 2009;12:444–54. 10.1017/S1368980008002401 18498676

[3] Hentze MW, Muckenthaler MU, Galy B, Camaschella C. Two to tango: regulation of Mammalian iron metabolism. Cell 2010;142:24–38. 20603012 10.1016/j.cell.2010.06.028

[4] Camaschella C Iron-deficiency anemia. N Engl J Med 2015;372: 1832–43. 25946282 10.1056/NEJMra1401038

[5] Wirth JP, Woodruff BA, Engle-Stone R, Namaste SM, Temple VJ, Petry N, Predictors of anemia in women of reproductive age: Biomarkers Reflecting Inflammation and Nutritional Determinants of Anemia (BRINDA) project. Am J Clin Nutr 2017;106:416S–27S. 28615262 10.3945/ajcn.116.143073 PMC5490645

[6] Baker RD, Greer FR, Committee on Nutrition American Academy of P. Diagnosis and prevention of iron deficiency and iron-deficiency anemia in infants and young children (0–3 years of age). Pediatrics 2010;126: 1040–50. 20923825 10.1542/peds.2010-2576

[7] Pfeiffer CM, Looker AC. Laboratory methodologies for indicators of iron status: strengths, limitations, and analytical challenges. Am J Clin Nutr 2017;106:1606S–14S. 29070545 10.3945/ajcn.117.155887 PMC5701713

[8] U.S. Centers for Disease Control and Prevention. Second national report on biochemical indicators of diet and nutrition in the U.S. population 2012. Atlanta (GA): National Center for Environmental Health; 2012.

[9] Joint World Health Organization/Centers for Disease Control and Prevention Technical Consultation on the assessment of iron status at the population level; 2005.

[10] Raiten DJ, Sakr Ashour FA, Ross AC, Meydani SN, Dawson HD, Stephensen CB, Inflammation and nutritional science for programs/policies and interpretation of Research evidence (INSPIRE). J Nutr 2015;145:1039S–108S. 25833893 10.3945/jn.114.194571 PMC4448820

[11] Borhani DW, Harrison SC. Crystallization and X-ray diffraction studies of a soluble form of the human transferrin receptor. J Mol Biol 1991;218: 685–9. 2023243 10.1016/0022-2836(91)90255-5

[12] Lawrence CM, Ray S, Babyonyshev M, Galluser R, Borhani DW, Harrison SC. Crystal structure of the ectodomain of human transferrin receptor. Science 1999;286:779–82. 10531064 10.1126/science.286.5440.779

[13] Turkewitz AP, Amatruda JF, Borhani D, Harrison SC, Schwartz AL. A high yield purification of the human transferrin receptor and properties of its major extracellular fragment. J Biol Chem 1988;263:8318–25. 3372526

[14] Richardson DR, Ponka P. The molecular mechanisms of the metabolism and transport of iron in normal and neoplastic cells. Biochim Biophys Acta 1997;1331:1–40. 9325434 10.1016/s0304-4157(96)00014-7

[15] Beguin Y Soluble transferrin receptor for the evaluation of erythropoiesis and iron status. Clin Chim Acta 2003;329:9–22. 12589962 10.1016/s0009-8981(03)00005-6

[16] Thorpe SJ, Heath A, Sharp G, Cook J, Ellis R, Worwood M. A WHO reference reagent for the Serum Transferrin Receptor (sTfR): international collaborative study to evaluate a recombinant soluble transferrin receptor preparation. Clin Chem Lab Med 2010;48:815–20. 20446759 10.1515/CCLM.2010.167

[17] Lebron JA, Bennett MJ, Vaughn DE, Chirino AJ, Snow PM, Mintier GA, Crystal structure of the hemochromatosis protein HFE and characterization of its interaction with transferrin receptor. Cell 1998; 93:111–23. 9546397 10.1016/s0092-8674(00)81151-4

[18] Jing SQ, Trowbridge IS. Identification of the intermolecular disulfide bonds of the human transferrin receptor and its lipid-attachment site. EMBO J 1987;6:327–31. 3582362 10.1002/j.1460-2075.1987.tb04758.x PMC553399

[19] Rutledge EA, Root BJ, Lucas JJ, Enns CA. Elimination of the O-linked glycosylation site at Thr 104 results in the generation of a soluble human-transferrin receptor. Blood 1994;83:580–6. 8286753

[20] Miller WG, Schimmel H, Rej R, Greenberg N, Ceriotti F, Burns C, IFCC working group recommendations for assessing commutability part 1: general experimental design. Clin Chem 2018;64:447–54. 29348163 10.1373/clinchem.2017.277525 PMC5832613

[21] Vesper HW, Miller WG, Myers GL. Reference materials and commutability. Clin Biochem Rev 2007;28:139–47. 18392124 PMC2282402

[22] Danilenko U, Vesper HW, Myers GL, Clapshaw PA, Camara JE, Miller WG. An updated protocol based on CLSI document C37 for preparation of off-the-clot serum from individual units for use alone or to prepare commutable pooled serum reference materials. Clin Chem Lab Med 2020;58:368–74. 31665109 10.1515/cclm-2019-0732 PMC7153737

[23] Budd JR, Weykamp C, Rej R, MacKenzie F, Ceriotti F, Greenberg N, IFCC working group recommendations for assessing commutability part 3: using the calibration effectiveness of a reference material. Clin Chem 2018;64:465–74. 29348164 10.1373/clinchem.2017.277558

[24] Nilsson G, Budd JR, Greenberg N, Delatour V, Rej R, Panteghini M, IFCC working group recommendations for assessing commutability part 2: using the difference in bias between a reference material and clinical samples. Clin Chem 2018;64:455–64. 29348165 10.1373/clinchem.2017.277541 PMC5835923

[25] Mei Z, Pfeiffer CM, Looker AC, Flores-Ayala RC, Lacher DA, Mirel LB, Serum soluble transferrin receptor concentrations in US preschool children and non-pregnant women of childbearing age from the National Health and Nutrition Examination Survey 2003–2010. Clin Chim Acta 2012;413:1479–84. 22705806 10.1016/j.cca.2012.05.022

[26] Braga F, Pasqualetti S, Frusciante E, Borrillo F, Chibireva M, Panteghini M. Harmonization status of serum ferritin measurements and implications for use as marker of iron-related disorders. Clin Chem 2022;68:1202–10. 35794075 10.1093/clinchem/hvac099

[27] Fraser CG. Biological variation: from principles to practice. Washington, DC: AACC Press; 2001.

[28] European Federation of Clinical Chemistry and Laboratory Medicine. The EFLM biological variation database. Available at: https://biologicalvariation.eu/ [Accessed August 2021].

[29] Carobene A, Aarsand AK, Guerra E, Bartlett WA, Coskun A, Diaz-Garzon J, European Biological Variation Study (EuBIVAS): within- and between-subject biological variation data for 15 frequently measured proteins. Clin Chem 2019;65:1031–41. 31171528 10.1373/clinchem.2019.304618

[30] Widjaja A, Morris RJ, Levy JC, Frayn KN, Manley SE, Turner RC. Within- and between-subject variation in commonly measured anthropometric and biochemical variables. Clin Chem 1999;45: 561–6. 10102917

[31] Miller WG, Myers GL. Commutability still matters. Clin Chem 2013;59: 1291–3. 23780914 10.1373/clinchem.2013.208785

[32] Harrison RL, Jarvis DL. Protein N-glycosylation in the baculovirus-insect cell expression system and engineering of insect cells to produce “mammalianized” recombinant glycoproteins. Adv Virus Res 2006;68: 159–91. 16997012 10.1016/S0065-3527(06)68005-6

