# Supplementary material for: Assessment of WHO 07/202 reference material and human serum pools for commutability and for the potential to reduce variability among soluble transferrin receptor assays
Source: Clin Chem Lab Med. Author manuscript; Available in PMC 2026 Jul 13. (PMC13361177; doi:10.1515/cclm-2022-1198)
Supplement: Supplement [file NIHMS2156445-supplement-Supplement.docx]

**Online Supplement**

**Assessment of WHO 07/202 reference material and human serum pools for commutability and for the potential to reduce variability among soluble transferrin receptor assays**

Alicia N. Lyle, Jeffery R. Budd, Victoria M. Kennerley, Bianca N. Smith, Uliana Danilenko, Christine M. Pfeiffer, Hubert W. Vesper

**Online Supplement Contents:**

**Expanded Materials and Methods.**

**Supplemental Table 1.** Freeze-thaw stability results for the WHO 07/202 sTfR reference material at two concentrations.

**Supplemental Table 2.** WHO 07/202 measurements (mg/L) from each MP and the percent difference from the WHO 07/202 target value (mg/L), before standardization.

**Supplemental Table 3.** Bias limits used as commutability criteria for each measurement procedure and the bias ranges derived for each material from the expanded measurement uncertainty using the difference in bias approach and following the principles described in IFCC Part 2.

**Supplemental Table 4.** Commutability results for WHO 07/202 RM dilutions, C37 serum pools, and non-C37 serum pools using the difference in bias approach and following the principles described in IFCC Part 2.

**Supplemental Table 5.** Median percent biases and SDs across all clinical samples for each MP and inter-measurement procedure bias range across MPs – before and after calibration to WHO 07/202 using the trimmed mean target and the calibration effectiveness approach following the principles described in IFCC Part 3.

**Expanded Materials and Methods.**

**Serum pools.** Human serum pools from BioIVT (Westbury, NY, USA) were generated used off-the-clot serum. According to information provided by BioIVT, blood was processed to generate non-C37 serum pools as follows: whole blood was drawn into a dry collection bag and spun at 5,000 x *g* for 10 min in a refrigerated centrifuge (5ºC), the supernatant was transferred into another bag and allowed to clot at room temperature for up to 48 hours, material was spun to serum at 5,000 x *g* for 20 minutes in a refrigerated centrifuge (4ºC). Serum materials went through 1 – 2 freeze thaw cycles.

**WHO 07/202 sTfR reference material.** WHO 07/202 stability was tested internally for the 0.8 mg/dL (11.7 nmol/L) and 2.17 mg/dL (30.3 nmol/L) dilutions. Two separate vials of each sTfR dilution were measured after each of two freeze-thaw cycles and compared to the initial sTfR measurement. To perform one freeze-thaw cycle, a sample vial is removed from -70°C and is thawed at room temperature with end-over-end rotation for 15 minutes, an appropriate volume of sample is transferred to a sample cup for measurements, and the remaining sample volume is refrozen at -70°C for 18 - 24 hours before the next thaw. Measurements were acquired using the Roche Tina-quant sTfR I assay on a Cobas^®^ c501 analyzer. The average recovery was 94.8%, which is within the ±10% recovery stated by the assay manufacturer.

| **Supplemental Table 1.** Freeze-thaw stability results for the WHO 07/202 sTfR reference material at two concentrations. | | | |
| --- | --- | --- | --- |
| **WHO 07/202 RM, 0.8 mg/dL** | **Initial  Measure (mg/dL)** | **One**  **Freeze-thaw (mg/dL)** | **Two**  **Freeze-thaws (mg/dL)** |
| Replicate 1 | 3.02 | 2.79 | 2.81 |
| Replicate 2 | 3.05 | 2.83 | 2.78 |
|  |  |  |  |
| Mean | 3.04 | 2.81 | 2.80 |
| SD | 0.02 | 0.03 | 0.02 |
| CV, % | 0.7% | 1.0% | 0.8% |
| Difference vs. Initial Measure, % | -- | -7.41 | -7.91 |
| **Recovery, %** | -- | 92.59 | 92.09 |
|  |  |  |  |
| **WHO 07/202 RM, 2.17 mg/dL** | **Initial  Measure (mg/dL)** | **One**  **Freeze-thaw (mg/dL)** | **Two**  **Freeze-thaws (mg/dL)** |
| Replicate 1 | 7.19 | 6.91 | 7.04 |
| Replicate 2 | 7.08 | 6.94 | 6.87 |
|  |  |  |  |
| Mean | 7.14 | 6.93 | 6.96 |
| SD | 0.08 | 0.02 | 0.12 |
| CV, % | 1.1% | 0.3% | 1.7% |
| Difference vs. Initial Measure, % | -- | -2.94 | -2.52 |
| **Recovery, %** | -- | 97.06 | 97.48 |

| **Supplemental Table 2.** WHO 07/202 measurements (mg/L) from each MP and the percent difference from the WHO 07/202 target value (mg/L), before standardization. | | | | | | |
| --- | --- | --- | --- | --- | --- | --- |
| **WHO 07/202 Values** | **S001** | **S002** | **S007** | **S008** | **S009** | **S010** |
| **mg/L** | **mg/L [%]** | **mg/L [%]** | **mg/L [%]** | **mg/L [%]** | **mg/L [%]** | **mg/L [%]** |
| 0.8 | 2.90 [263] | 1.42 [78.5] | 2.51 [214] | 0.96 [20.0] | 0.90 [12.5] | 0.70 [-12.5] |
| 1.1 | 3.63 [230] | 1.83 [66.4] | 3.47 [215] | 1.22 [10.9] | 1.14 [3.6] | 0.89 [-19.1] |
| 1.35 | 4.54 [236] | 2.35 [74.1] | 4.30 [219] | 1.47 [8.9] | 1.39 [3.0] | 1.06 [-21.5] |
| 1.8 | 5.92 [229] | 3.20 [77.8] | 6.34 [252] | 1.91 [6.1] | 1.83 [1.7] | 1.40 [-22.2] |
| 2.17 | 6.98 [222] | 3.84 [77.0] | 7.50 [246] | 2.26 [4.1] | 2.17 [0] | 1.77 [-18.4] |
| 2.7 | 8.65 [220] | 4.91 [81.9] | 10.39 [285] | 2.69 [-0.4] | 2.58 [-4.4] | 2.29 [-15.2] |

| **Supplemental Table 3.** Bias limits used as commutability criteria for each measurement procedure and the bias ranges derived for each material from the expanded measurement uncertainty using the difference in bias approach and following the principles described in IFCC Part 2. | | | | | | |
| --- | --- | --- | --- | --- | --- | --- |
| **Lab ID** | **S001** | **S002** | **S007** | **S008** | **S009** | **S010** |
| **Bias Limits** | -0.68 – 2.57 | -0.77 – 2.48 | -0.49 – 2.76 | -1.55 – 1.70 | -1.64 – 1.61 | -1.69 – 1.56 |
| **Material** | **Bias Ranges around the Mean Bias** | | | | | |
| WHO 07/202, 0.8 mg/L | 0.90 – 1.55 | 0.19 – 0.83 | 0.70 – 1.45 | -0.20 – 0.44 | -0.27 – 0.38 | -0.53 – 0.12 |
| WHO 07/202, 1.1 mg/L | 0.88 – 1.53 | 0.20 – 0.85 | 0.78 – 1.53 | -0.21 – 0.44 | -0.27 – 0.37 | -0.52 – 0.13 |
| WHO 07/202, 1.35 mg/L | 0.92 – 1.57 | 0.27 – 0.91 | 0.81 – 1.57 | -0.20 – 0.44 | -0.27 – 0.38 | -0.53 – 0.12 |
| WHO 07/202, 1.8 mg/L | 0.91 – 1.56 | 0.30 – 0.94 | 0.93 – 1.68 | -0.21 – 0.43 | -0.26 – 0.39 | -0.53 – 0.12 |
| WHO 07/202, 2.17 mg/L | 0.89 – 1.54 | 0.30 – 0.94 | 0.90 – 1.65 | -0.24 – 0.41 | -0.27 – 0.27 | -0.48 – 0.17 |
| WHO 07/202, 2.7 mg/L | 0.91 – 1.56 | 0.34 – 0.99 | 1.04 – 1.79 | -0.26 – 0.39 | -0.30 – 0.35 | -0.42 – 0.23 |
| C37, Low | 0.79 – 1.09 | 0.71 – 0.99 | 0.79 – 1.29 | -0.08 – 0.21 | -0.18 – 0.11 | -0.19 – 0.12 |
| C37, Medium | 0.71 – 1.00 | 0.70 – 0.98 | 0.77 – 1.27 | -0.07 – 0.21 | -0.16 – 0.13 | -0.21 – 0.09 |
| C37, High | 0.94 – 1.24 | 0.76 – 1.03 | 0.94 – 1.44 | -0.10 – 0.19 | -0.16 – 0.13 | -0.18 – 0.12 |
| non-C37, Low | 0.53 – 1.23 | 0.51 – 1.20 | 0.92 – 1.68 | -0.26 – 0.43 | -0.39 – 0.30 | -0.39 – 0.30 |
| non-C37, Medium | 0.57 – 1.26 | 0.52 – 1.21 | 0.69 – 1.45 | -0.28 – 0.41 | -0.38 – 0.31 | -0.38 – 0.31 |
| non-C37, High | 0.64 – 1.34 | 0.45 – 1.14 | 0.84 – 1.59 | -0.23 – 0.46 | -0.31 – 0.38 | -0.38 – 0.17 |

| **Supplemental Table 4.** Commutability results for WHO 07/202 RM dilutions, C37 serum pools, and non-C37 serum pools using the difference in bias approach and following the principles described in IFCC Part 2. | | | | | | |
| --- | --- | --- | --- | --- | --- | --- |
|  | **Commutability Results** | | | | | |
| **Material** | **S001** | **S002** | **S007** | **S008** | **S009** | **S010** |
| WHO 07/202, 0.8 mg/L | C | C | C | C | C | C |
| WHO 07/202, 1.1 mg/L | C | C | C | C | C | C |
| WHO 07/202, 1.35 mg/L | C | C | C | C | C | C |
| WHO 07/202, 1.8 mg/L | C | C | C | C | C | C |
| WHO 07/202, 2.17 mg/L | C | C | C | C | C | C |
| WHO 07/202, 2.7 mg/L | C | C | C | C | C | C |
| C37, Low | C | C | C | C | C | C |
| C37, Medium | C | C | C | C | C | C |
| C37, High | C | C | C | C | C | C |
| non-C37, Low | C | C | C | C | C | C |
| non-C37, Medium | C | C | C | C | C | C |
| non-C37, High | C | C | C | C | C | C |
| C = Commutable, N = Noncommutable, I = Indeterminate | | | | | | |

| **Supplemental Table 5.** Median percent biases and SDs across all clinical samples for each MP and inter-measurement procedure bias range across MPs – before and after calibration to WHO 07/202 using the trimmed mean target and the calibration effectiveness approach following the principles described in IFCC Part 3. | | | | | | |
| --- | --- | --- | --- | --- | --- | --- |
|  | **S001** | **S002** | **S007** | **S008** | **S009** | **S010** |
| **Before Standardization** |  |  |  |  |  |  |
| Median Bias Before Standardization, % | 46.4 | 35.3 | 72.4 | -37.5 | -43.4 | -44.8 |
| SD Before Standardization, % | 6.4 | 3.0 | 34.7 | 2.4 | 1.9 | 4.1 |
| **IMPBR Before, %** | **117.2** |  |  |  |  |  |
|  |  |  |  |  |  |  |
| **After Calibration to WHO 07/202** |  |  |  |  |  |  |
| Median Bias After Standardization, % | -26.0 | 29.1 | -10.7 | -1.5 | -5.9 | 16.8 |
| SD After Standardization, % | 6.1 | 5.2 | 12.4 | 5.7 | 5.2 | 5.2 |
| **IMPBR After, %** | **55.0** |  |  |  |  |  |
